# The advantages of preoperative 3D reconstruction over 2D-CT in thoracoscopic segmentectomy

**DOI:** 10.1007/s13304-024-01965-6

**Published:** 2024-09-29

**Authors:** Hao He, Peiyuan Wang, Hang Zhou, Wenwei Wei, Junpeng Lin, Yujie Chen, Feng Wang, Shuoyan Liu

**Affiliations:** 1 Department of Thoracic Oncology Surgery, Fujian Cancer Hospital, Clinical Oncology School of Fujian Medical University, 420 Fu Ma Road, Jin’an District, Fuzhou, China; 2 Fujian Key Laboratory of Translational Cancer Medicine, Fuzhou, China; 3 Fujian Provincial Key Laboratory of Tumor Biotherapy, Fuzhou, China

**Keywords:** Advantages, Three-dimensional reconstruction (3D-RE), Two-dimensional computed tomography (2D-CT), Thoracoscopic segmentectomy

## Abstract

Performing a pulmonary segmentectomy is a complex process, with precise localization of pulmonary nodules and recognition of intraoperative anatomical variations posing significant challenges. This study aims to assess the advantages of preoperative three-dimensional reconstruction (3D-RE) in thoracoscopic segmentectomy. The study, at Fujian Medical University Cancer Hospital, analyzed data from segmentectomy patients from January 2016 to February 2022. It compared 3D-RE and two-dimensional computed tomography (2D-CT) preoperative scans, focusing on perioperative complications within30 days to identify any differences. This investigation encompassed a total of 265 instances, with 148 belonging to the 3D-RE group and 117 aligned with the 2D-CT group. The 3D-RE group showed reduced intraoperative blood loss and shorter postoperative hospital stays (*P* < 0.001). They also had higher rates of lymph node sampling and combined subsegmentectomy and segmentectomy procedures (*P* < 0.01). Postoperative complications, particularly pneumonia and lung fistula, were lower in the 3D-RE group (*P* = 0.041). The rates of minimally invasive adenocarcinoma (MIA) and invasive adenocarcinoma (IAC) were significantly higher in the 3D-RE group, while adenocarcinoma in situ (AIS) and benign cases were less common (*P* = 0.006). Surgical duration, chest tube duration, chest drainage volume, surgery complexity, and pathological diagnoses showed no significant differences between the groups. Utilization of preoperative 3D-RE holds potential to minimize both intraoperative and postoperative complications, thereby enhancing the safety and feasibility of undertaking segmentectomy procedures.

## Introduction

The freshly published Global Cancer Statistics 2020 indicates that lung cancer ranks second in terms of diagnosis prevalence and first in terms of cancer-induced mortality for the year 2020, constituting roughly 11.4% of cancer diagnoses and contributing to 18.0% of deaths [1]. Lately, the utilization of low-dose chest computed tomography (CT) witnessing a rise, consequently escalating the detection rate of minuscule pulmonary nodules. The standard procedure for patients with stage I non-small cell lung cancer (NSCLC) is lobectomy and mediastinal lymph node sampling or none.

The ongoing exploration of segmentectomy is unearthing mounting evidence consolidating the equivalency of its prognosis to lobectomy in the context of early-stage lung cancer, with the former displaying superior preservation of lung function [2–6]. Compared to wedge resection, segmentectomy ensures sufficient tumor staging alongside the achievement of surgical margin and lymph node sampling, thereby significantly outperforming the former in prognosis [7–9].Hence, lobectomy-intolerant patients diagnosed with early lung cancer, benign lesions, and metastatic tumors should prioritize segmentectomy.

Conversely, pulmonary segmentectomy embodies a highly challenging procedure, particularly when considering the accurate positioning of pulmonary nodules and therein identification of intraoperative anatomical variation. Preoperative 2D-CT image accuracy in detailing the intricate anatomical structures of lungs such as arteries, bronchi and veins falls short, inherently escalating the risk of segmentectomy precision. As medical imaging technology advances, 3D-RE is gradually seeing application in thoracoscopic segmentectomy. Its abilities to precisely locate pulmonary nodules, estimate the resection's scope and determine the intersegmental plane's position, as well as detect variant bronchi and blood vessels, render surgery more accurate and safe. Hence, the pivotal role that preoperative 3D-RE plays in accurate segmentectomy becomes clear [10–14].

In this study, we looked back over the in-depth clinical characteristics of segmentectomy patients from our department and juxtaposed preoperative 3D-RE with 2D-CT in thoracoscopic surgery. The purpose of this article lies in assessing the impact of preoperative 3D-RE in thoracoscopic segmentectomy, thereby offering a point of reference for clinical application.

## Material & methods

### Patients

In this retrospective analysis, patients who underwent anatomical segmentectomy via video-assisted thoracoscopic surgery (VATS) at the Fujian Cancer Hospital’s Department of Thoracic Surgery, from January 2016 through February 2022, were considered. All the surgical procedures were uniformly performed by one designated thoracic surgeon who had performed over 300 lung segmentations.

### Inclusion criteria

(I) the feasibility of the segmentectomy, as dictated by a safe resection margin which was larger than 2 cm or the diameter of the pulmonary nodule;
(II) the execution of all anatomical segmentectomies via uniport or two-port VATS;
(III) the necessity of mediastinal lymph node sampling or none for malignant pulmonary nodules;
(IV) the allowance of benign or malignant pulmonary nodules for inclusion;
(V) no restrictions based on age and gender.

### Exclusion criteria

(I) the presence of pulmonary nodules located centrally within the lobe that couldn't be targeted with segmentectomy;
(II) localization within the middle lobe;
(III) diagnosis of severe emphysema;
(IV) presence of severe whole-chest adhesions;
(V) history of lung surgery;
(VI) occurrence of distant metastasis;
(VII) insufficiency of essential clinical data.

**Fig. 1:**
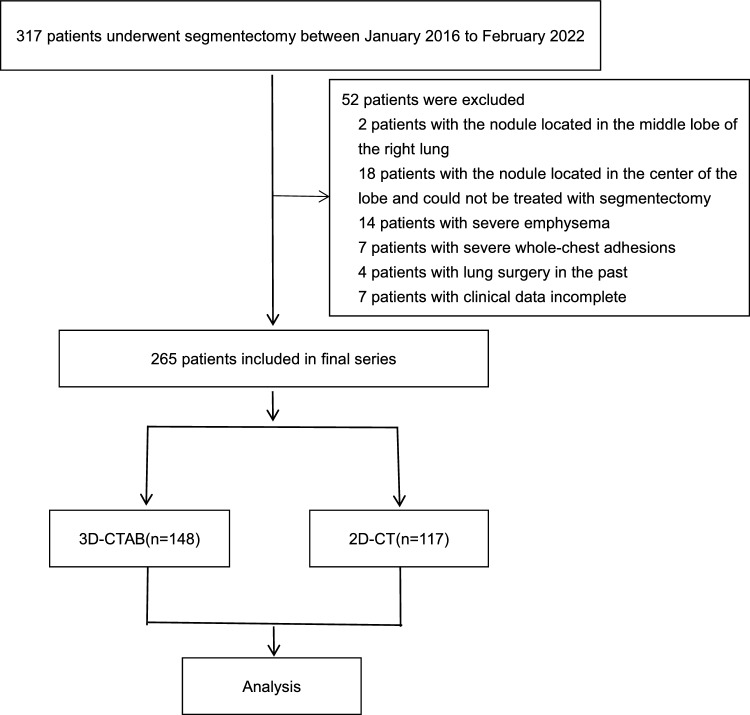
Study flowchart

Upon applying these eligibility parameters, a total of 264 patients were listed for the retroactive analysis (Fig. 1).

### 3D-RE

Employing a 256-channel Multi-detector Computed Tomography (MDCT, General Electric Healthcare, Boston, MA, USA), lung imaging was executed with a slice thickness specification of 0.625 mm. As part of the procedure, an intravenous injection of 35 ml contrast medium was administered at a velocity of 5 ml/s, instantly succeeded by 20 ml of normal saline, utilizing an automatic injector to amplify vessel enhancement. The acquired thin-slice two-dimensional CT images, in Digital Imaging and Communications in Medicine (DICOM) format, were transitioned to dedicated software (IQQA-Lung, EDDA Technology, Princeton, NJ, USA). This software facilitated the reconstruction of a three-dimensional model encompassing the bronchial and vessel system and the coordinates of the nodule. Potential errors and perceived defects predominantly affecting the distal bronchial and vessel imaging were subsequently manually rectified.

### Case grouping

3D-RE required an additional cost, so we do it on a voluntary basis. The patients were categorized into two groups based on the utilization of 3D-RE: the 3D-RE group and the 2D-CT group.

### The 3D-RE group

#### Preoperative surgical simulation

**Fig. 2:**
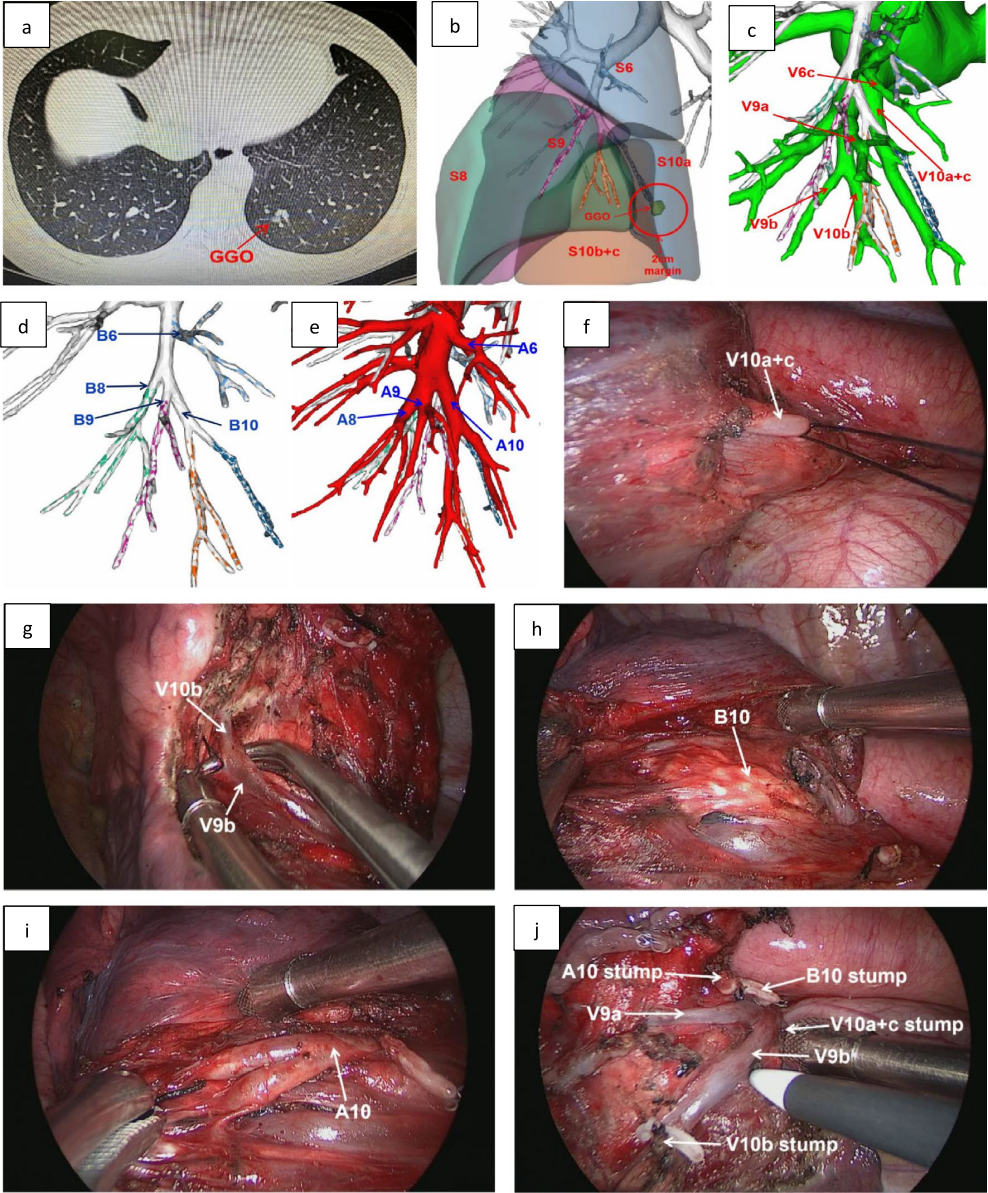
The CT scan and 3D-CTAB of a 48-year-old female patient. One GGO was found in the left lower lobe. According to the 3D-CTAB, anatomical segmentectomy with left S10 was performed; **a** the CT scan showed that a GGO was located in the left lobe (S10); **b** according to the results of 3D-CTAB and the safe margin of 2 cm, the confirmed target lung segment was LS10; **c** 3D-CTAB showed pulmonary vein anatomy; **d** 3D-CTAB showed the pulmonary bronchial anatomy; **e** 3D-CTAB showed the pulmonary artery anatomy; **f** V10a + c was showed in the operation; **g** V9b and V10b was showed in the operation; **h** B10 was showed in the operation; **i** A10 was showed in the operation; **j** the surgical wound after LS10 segmentectomy

Utilising 3D-RE imaging, we can identify the precise location of the nodules and their respective lung segment. Following this determination, we verified the target segmental bronchus, arteries, segmental veins, and intersegmental veins, subsequently formulating the surgical approach. Furthermore, it's imperative to highlight that the quantification of the pulmonary nodule's maximum diameter was conducted via the CT lung window. An essential stipulation for resection margins was that they must be a minimum of 2 cm or exceed the maximum diameter of the pulmonary nodule, as illustrated by Fig. 2.

### Surgical procedures

Utilizing general anesthesia in conjunction with double-lumen endotracheal intubation, all patients underwent a one-lung ventilation procedure on the contralateral side and were positioned laterally. The ensuing surgical procedures were executed by one seasoned thoracic surgeon, with the assistance of either uniport or two-port video assisted thoracic surgery.

The single-port surgery entailed establishing a 4 cm utility incision within the anterior axillary line through the fourth intercostal space (ICS). In contrast, the two-port surgery procedure additionally incorporated a 10 mm trocar into the midaxillary line inside the ICS to enable the access of a 30-degree thoracoscope. In alignment with the predetermined surgical simulation, the intended arteries, veins, and bronchi were severed, while the intersegmental veins were preserved. The differentiation of the targeted and non-targeted segment borders were subsequently identified using the “inflation-deflation” approach. The interface between the inflation and deflation was dissected along the intersegmental veins towards the outer one third of the pulmonary parenchyma using ultrasonic scalpels. The remaining pulmonary parenchyma was then resected using staplers. Utilizing an ultrasonic knife, the pulmonary parenchyma was separated along the inflation-deflation line and the intersegmental veins from the segment hilum to the outer one-third of the lung tissue. Linear cutting staplers were then employed for the removal of remaining lung tissue. A pulmonary leakage test was conducted post-procedure to assess lung durability. In cases of evident air leakage, suturing with a 3-0 or 4-0 Prolene suture was performed. If air leakage was minimal, the wound was covered with hemostatic materials for closure. In operative scenarios involving malignant nodules, intra-pulmonary and mediastinal lymph nodes were isolated for examination via intraoperative frozen section testing. Signs of malignancy within the lymph nodes prompted the execution of a lobectomy along with a systematic lymph node dissection.

The surgical approach encompassed segmentectomy, subsegmentectomy, and combined segmentectomy. Segmentectomy was characterized as excision of a single lung segment. Subsegmentectomy was described as excision of a solitary lung subsegment. Combined segmentectomy was typified as resection of a minimum of two subsegments, a segment and a subsegment, or two or more pulmonary segments.

As delineated in a previous study [3], the categorization of segmentectomy was orchestrated into simple and intricate segmentectomy, dependent on the number of intersegmental planes excised. In instances when a solo intersegmental plane was subject to resection, it was classified as a simple segmentectomy, evidenced in the superior segment of the right or left lower lobe (S6), basal segment, left superior, and the lingular segment. The remaining variants were organized under complex segmentectomy.

### The 2D-CT group

Reliant on empirical knowledge and preoperative thin-section 2D-CT sans, the thoracic surgeon delineated the location of pulmonary nodules and the designated pulmonary segment, alongside the vascular and bronchial structures intended for resection. Ancillary procedures remained congruent with those executed in the 3D-RE group.

### Statistical analysis

Analytical procedures of all accrued clinical data were undertaken utilizing SPSS (IBM SPSS Statistics for Windows, Version 22.0, IBM Corp., Armonk, NY, USA). Continuous data presentations adhered to the mean ± standard deviation (SD) model, and the *t*-test or Wilcoxon test enacted comparisons of quantitative continuous data. Usage of Chi-square or Fishers exact tests was prompted by the necessity for categorical variables. A *P* value of < 0.05 was the threshold for statistical significance.

## Results

### Clinical characteristics of the patients

In this investigation, a total of 265 subjects were considered eligible, including 148 within the 3D-RE cohort and 117 falling into the 2D-CT group. Table 1 provides comprehensive delineation of the clinical profiles for both study groups.

**Table 1:** presents the clinical characteristics of each group, with the data reported as n (%) and means (SD) unless otherwise specified

Variable	3D-CTAB(148)	2D-CT(117)	t/χ2	P
Age(years)(SD)	56.0 ± 10.7	54.5 ± 11.8	1.047	0.296
Gender			1	0.981
Male	66(44.6%)	52(44.4%)		
Female	82(55.4%)	65(55.6%)		
Smoking			0.030	0.862
Yes	13(8.8%)	11(9.4%)		
No	135(91.2%)	106(90.6%)		
Maximum lesion diameter(mm) (SD)	12.54 ± 5.30	13.88 ± 6.67	-1.823	0.069
Nodule location			0.996	0.802
LUL	48(32.4%)	32(27.4%)		
LLL	31(20.9%)	25(21.4%)		
RUL	38(25.7%)	35(29.9%)		
RLL	31(20.9%)	25(21.4%)		
Pulmonary function				
FEV1	2.59 ± 0.61	2.47 ± 0.54	1.690	0.092
FEV1 (%)	105.12 ± 89.78	90.80 ± 14.01	1.650	0.100
Hypertension			0.000	0.990
Yes	29(19.6%)	23(19.7%)		
No	119(80.4%)	94(80.3%)		
Diabetes			0.590	0.442
Yes	14(9.5%)	8(6.8%)		
No	134(90.5%)	109(93.2%)		
COPD			0.578	0.447
Yes	18(12.2%)	18(15.4%)		
No	130(87.8%)	99(84.6%)		

### Intraoperative and postoperative data

Thoracoscopic segmentectomies were successfully executed for all individuals, circumventing any need for conversion to thoracotomy or lobectomy. It should be noted that within the 2D-CT arm, five subjects underwent combined segmentectomy due to varying issues, one being an inadequate resection margin, two due to missection of the pulmonary artery and another two due to bronchial miscuts. Importantly, lymph nodes from all individuals showed no sign of pathology, and the surgical margins were within acceptable limits according to the established surgical parameters.

The 3D-RE group exhibited a decrease in intraoperative blood loss and shorter hospital stays post-surgery relative to their 2D-CT counterparts (77.5 ± 29.0 mL versus 120 ± 68.5 mL, and 5.7 ± 3.5 days versus 7.3 ± 3.6 days respectively), with statistical significance denoted (P < 0.05). Lymph node sampling was also notably higher within the 3D-RE group, constituting 89.2% in comparison to 78.6% in the 2D-CT group. Regarding surgical modalities, a significantly higher occurrence of subsegmentectomy and combined segmentectomy was noted in the 3D-RE group (8.8% and 48.6% respectively) when juxtaposed with the 2D-CT group (0% and 42.7% respectively). Moreover, the postoperative complications in the 3D-RE cohort were markedly lower (8.1% vs. 16.2%), with particular reference to incidences of pneumonia and lung fistula. Lung fistula was defined as a leak of air in the lungs lasting more than 5 days. Stratified analysis carried out among adenocarcinoma subjects demonstrated that MIA was significantly higher within the 3D cohort (53.7% compared to 38.8%), while AIS was less prevalent (9.1% versus 24.7%). Contrasts between the groups in terms of average operative duration, length of chest tube placement, total chest drainage, level of surgical difficulty or pathological diagnosis did not show a significant statistical difference (P > 0.05)—details depicted in Table 2.

**Table 2:** Intraoperative and postoperative data of the two group

Variable	3D-CTAB(148)	2D-CT(117)	t/χ^2^	P
Average operative duration (minutes)	170.5 ± 47.4	176 ± 52.3	– 0.925	0.356
Intraoperative blood loss(mL)	77.5 ± 29.0	120 ± 68.5	– 6.336	0.000
The duration of chest tube placement (days)	3.2 ± 3.4	3.8 ± 3.3	– 1.488	0.138
Postoperative hospital stays(days)	5.7 ± 3.5	7.3 ± 3.6	– 3.524	0.001
Postoperative complications	12(8.1%)	19(16.2%)	4.182	0.041
Pneumonia	2(1.4%)	3(2.6%)		
Atelectasis	0(0%)	2(1.7%)		
Chylothorax	2(1.4%)	0(0%)		
Lung fifistula	10(6.8%)	16(13.7%)		
Reinsertion of chest drain	4(2.7%)	3(2.6%)		
Mediastinal lymph node			5.568	0.018
None	16(10.8%)	25(21.4%)		
Sampling	132(89.2%)	92(78.6%)		
Total chest drainage (mL)	835.9 ± 712.1	957.9 ± 605.2	– 1.479	0.140
Mode of surgery			13.651	0.001
Segmentectomy	63(42.6%)	67(57.3%)		
Subsegmentectomy	13(8.8%)	0(0%)		
Combined Segmentectomy	72(48.6%)	50(42.7%)		
surgical difficulty level			0.998	0.318
simple segmentectomy	63(42.6%)	57(48.7%)		
complex segmentectomy	85(57.4%)	60(51.3%)		
Pathological diagnosis			5.816	0.121
Adenocarcinoma	121(81.8%)	85(72.6%)		
Squamous cell Carcinoma	0(0%)	2(1.7%)		
Benign	27(18.2%)	29(24.8%)		
Carcinoid	0(0%)	1(0.9%)		
Adenocarcinoma			10.172	0.006
AIS	11(9.1%)	21(24.7%)		
MIA	65(53.7%)	33(38.8%)		
IAC	45(37.2%)	31(36.5%)		

## Discussion

The 1995 LCSG study established lobectomy as the standard surgical method for T1N0 NSCLC [15]. Segmentectomy's efficacy as an alternative to lobectomy for early NSCLC is debated. The JCOG0802 trial compared segmentectomy with lobectomy for ≤ 2 cm peripheral NSCLC. Segmentectomy had higher air leakage [3] but superior 5-year survival rates with less decline in FEV1 at 6 and 12 months. Segmentectomy is recommended for patients with small peripheral NSCLC [4]. The CALGB 140503 Trial confirmed segmentectomy's non-inferiority to lobectomy with advantages in lung function preservation [2, 6, 16]. NCCN guidelines suggest segmentectomy or wedge resection for patients with poor pulmonary reserve or comorbidities contraindicating lobectomy and nodules meeting specific criteria [17]. Segmentectomy is recognized as a viable option for early NSCLC treatment [18].

Nevertheless, it should be noted that segmentectomy posed inherent challenges as a surgical procedure. To commence, a key hurdle involved precise localization of the pulmonary nodule and identification of the targeted segment. This not only ensured an adequate surgical margin but also facilitated efficient lesion detection, ultimately reducing operation time. Additionally, the anatomy of lung segments is considerably more intricate compared to the structure of lung lobes. There exists a wide range of anatomical variations pertaining to bronchial and vascular structures, thereby necessitating thoracic surgeons' comprehensive familiarity with the specific lung anatomy of each patient.

Previous practices involved the utilization of 2D-CT for locating pulmonary nodules and identifying pulmonary anatomical structures. However, this approach presented challenges in accurately discerning the anatomical structures of target segments, pulmonary arteries, veins, and bronchi. Consequently, there was a higher risk of misplanning the target lung segment. Inadvertent damage to the pulmonary vessels or bronchi during surgery could necessitate combined segmentectomy or lobectomy, potentially resulting in severe consequences. Notably, several studies have demonstrated that more than one-third of p-T1N0M0 NSCLC tumors extend beyond a single segment [19]. Reliance solely on 2D-CT for locating pulmonary nodules in such cases would make it significantly more arduous to determine the targeted segment and ensure adequate surgical margins. In the 2D-CT group of the present study, insufficient resection margin was observed in one case, mis-cutting of the pulmonary artery occurred in two cases, and mis-cutting of the bronchus occurred in two cases, necessitating expanded surgery and combined segmental resection. Conversely, no similar occurrences were reported in the 3D-RE group. Additionally, we observed a higher incidence of pneumonia and atelectasis in the 2D-CT group, possibly due to accidental damage to the bronchi or vessels during surgery.

The advancement of medical imaging technology has facilitated the gradual incorporation of 3D-RE technology in thoracoscopic segmentectomy procedures. 3D-RE enables the precise restoration of the lung's true anatomical structure with remarkable clarity. It offers the capability to accurately locate nodules, estimate resection ranges, determine target segments, and provide clear visualization of lung segment anatomical structures along with diverse bronchial and vascular anatomical variations. In light of these advantages, our medical center has also adopted 3D-RE technology for analyzing lung anatomical structures, uncovering rare anatomical variations [20–22]. Leveraging preoperative 3D-RE data, personalized and precise pulmonary segment resection plans are developed for each patient, and intraoperative navigation is performed. This integrated approach enhances surgical accuracy and safety throughout the procedure [12, 14, 23, 24].In our study, the 3D-RE group showed a higher lymph node sampling rate and fewer surgery-related complications. This further demonstrates the advantages of the 3D group, leading to the decision not to conduct a matching analysis. Moreover, we believe that the 3D-RE group displayed clear and precise delineation between target and non-target segments through preoperative 3D planning, thereby reducing the incidence of air leakage. Additionally, the 3D-RE group underwent more subsegmentectomy or combined segmentectomy procedures than the 2D-CT group, which are technically challenging and involve a larger surgical injury surface. Nevertheless, patients in the 3D-RE group experienced shorter surgical times, less blood loss, fewer postoperative complications, and shorter hospital stays, highlighting the benefits of 3D-RE.

### Limitation

The present investigation was subject to certain constraints. Initially, the failure to achieve maximum deep aspiration during a CT scan could engender inadequate expansion, leading to an incomplete bronchial reconstruction. Secondary to this, there is the likelihood that smaller bronchi, arteries or veins may have been overlooked, generating potential bias in our findings. Finally, the limits of the collective sample size solicits the need for an extended, multi-center investigation to fortify these outcomes.

## Conclusion

Engaging in preoperative 3D-RE offers substantial benefits in discerning pulmonary nodule locations and identifying anatomical lung variations prior to surgery. This technology permits comprehensive strategic planning and intraoperative navigation to facilitate an accurate process of pulmonary segmentectomy, thus decreasing risks during and post-operation. For pulmonary segmentectomy, the application of preoperative 3D-RE is both safe and practical.

## Data Availability

The data will be made available on reasonable request.

## Abbreviations

3D-RE: Three-dimensional reconstruction
2D-CT: Two-dimensional computed tomography
COPD: Chronic obstructive pulmonary disease
MIA: Minimally invasive adenocarcinoma
IAC: Invasive adenocarcinoma
AIS: Adenocarcinoma in situ
CT: Computed tomography
NSCLC: Non-small cell lung cancer
VATS: Video-assisted thoracoscopic surgery
DICOM: Digital Imaging and Communications in Medicine
ICS: Intercostal space
SD: Standard deviation
LCSG: Lung Cancer Study Group
NCCN: National Comprehensive Cancer Network
GGO: Ground-glass opacity
LUL: Left upper lobe
LLL: Left lower lobe
RUL: Right upper lobe
RLL: Right lower lobe
